# Common processes in pathogenesis by fungal and oomycete plant pathogens, described with Gene Ontology terms

**DOI:** 10.1186/1471-2180-9-S1-S7

**Published:** 2009-02-19

**Authors:** Shaowu Meng, Trudy Torto-Alalibo, Marcus C Chibucos, Brett M Tyler, Ralph A Dean

**Affiliations:** 1 Fungal Genomics Laboratory, Center for Integrated Fungal Research, North Carolina State University, Raleigh, NC 27695, USA; 2 Virginia Bioinformatics Institute, Virginia Polytechnic and State University, Blacksburg, VA 24061, USA; 3 Current address: Hayes Laboratory, Lineberger Comprehensive Cancer Center, School of Medicine, CB# 7295, University of North Carolina at Chapel Hill, Chapel Hill, NC 27599-7295, USA; 4 Current address: Institute for Genome Sciences, University of Maryland School of Medicine, Baltimore, MD 21201, USA

## Abstract

Plant diseases caused by fungi and oomycetes result in significant economic losses every year. Although phylogenetically distant, the infection processes by these organisms share many common features. These include dispersal of an infectious particle, host adhesion, recognition, penetration, invasive growth, and lesion development. Previously, many of these common processes did not have corresponding Gene Ontology (GO) terms. For example, no GO terms existed to describe processes related to the appressorium, an important structure for infection by many fungi and oomycetes. In this mini-review, we identify common features of the pathogenic processes of fungi and oomycetes and create a pathogenesis model using 256 newly developed and 38 extant GO terms, with an emphasis on the appressorium and signal transduction. This set of standardized GO terms provides a solid base to further compare and contrast the molecular underpinnings of fungal and oomycete pathogenesis.

## Common pathogenesis programs of fungi and oomycetes

Oomycetes, although phylogenetically very distant, share many common morphological and physiological features with the true fungi [1-3]. For example, they have similar filamentous, branching, indeterminate bodies, and they acquire nutrition by secreting digestive enzymes and then absorbing the resultant breakdown products. More importantly, fungi and oomycetes share a unique capability compared with other microbial pathogens, namely that they are able to breach cuticles of host plants and establish infection rapidly [4]. Consequently, both are causal agents of many destructive plant diseases and are responsible for significant economic losses every year.

**Figure 1:**
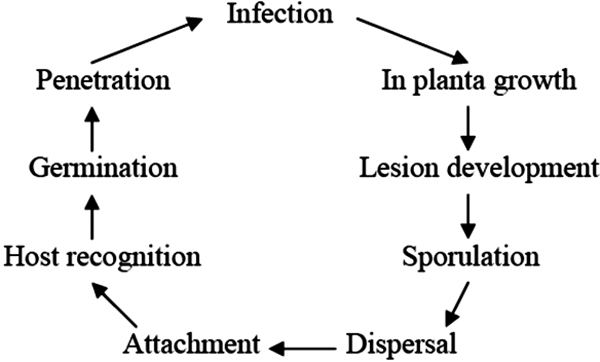
A generalized diagram displaying infection and disease cycle caused by fungi and oomycetes.

**Figure 2:**
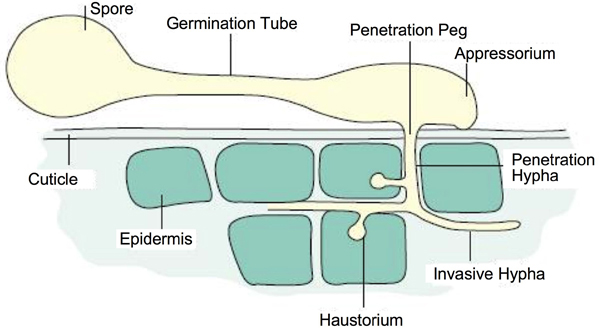
**The infection process in fungal and oomycete pathogens**. Modified by permission from Schumann, G. L., 1991, Plant diseases: Their biology and social impact, American Phytopathological Society, St. Paul, MN.

**Figure 3:**
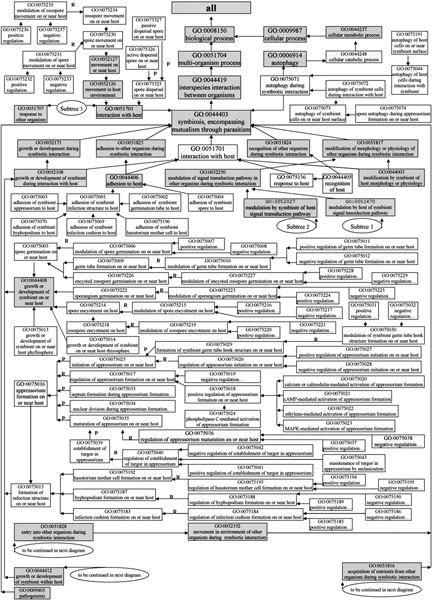
**Gene Ontology terms for processes related to infection and disease (Part 1)**. Subtree 1 and 2 are depictured in Figure 5, and Subtree 3 is depictured in Figure 6. Shaded boxes indicate pre-existing GO terms, and unshaded boxes represent GO terms developed under the PAMGO project. "R" indicates "regulates relationship", "P" indicates "part of relationship", and null indicates "is a relationship" (see the Gene Ontology website at  for further information).

**Figure 4:**
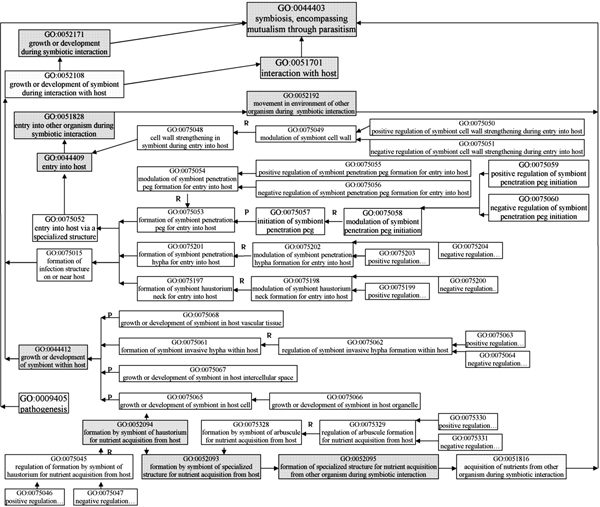
**Gene Ontology terms for processes related to infection and disease (Part 2)**. Shaded boxes indicate pre-existing GO terms, and unshaded boxes represent GO terms developed under the PAMGO project. "R" indicates "regulates relationship", "P" indicates "part_of relationship", and null indicates "is_a relationship" (see the Gene Ontology website at  for further information).

**Figure 5:**
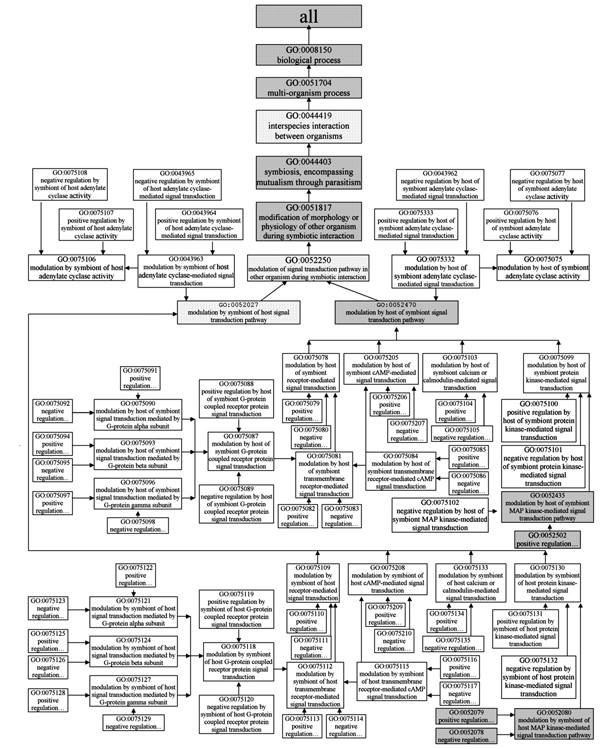
**Gene Ontology terms for signal transduction processes related to infection and disease (Part 1)**. Subtree 1 consists of GO terms intending to annotate host gene products that stimulate signal transduction in symbiont. Subtree 2 represents the opposite perspective of Subtree 1. Shaded boxes indicate pre-existing GO terms, and unshaded boxes represent GO terms developed under the PAMGO project.

**Figure 6:**
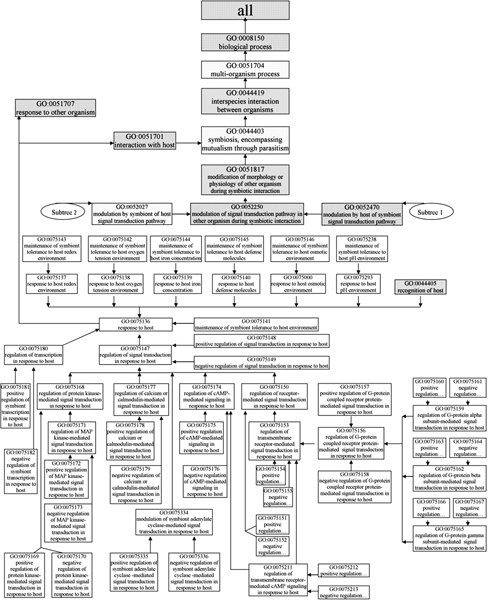
**Gene Ontology terms for signal transduction processes related to infection and disease (Part 2)**. Subtree 3 consists of GO terms intending to annotate symbiont gene products that stimulate signal transduction in symbiont in response to host. Shaded boxes indicate pre-existing GO terms, and unshaded boxes represent GO terms developed under the PAMGO project.

In this review, we summarize common mechanisms of pathogenesis displayed by oomycetes and fungi. Pathogenesis by a fungus or oomycete is a complex process. Briefly, it includes the following steps: dispersal and arrival of an infectious particle (usually a spore of some kind) in the vicinity of the host, adhesion to the host, recognition of the host (which may occur prior to adhesion), penetration into the host, invasive growth within the host, lesion development in the host, and finally production of additional infectious particles [5,6] (see Figures 1, 2). In order to describe the entire process, we formulate a description of pathogenesis using standardized terms from the Gene Ontology (GO), including 256 new terms developed by members of the PAMGO (Plant-Associated Microbe Gene Ontology) consortium , an official interest group of the GO Consortium, as well as 38 extant GO terms that are placed in shaded boxes in Figures 3, 4, 5, 6.

All of the 256 new terms are placed within GO under the node "GO ID 0044403 symbiosis, encompassing mutualism through parasitism" (note the original, broad definition of "symbiosis" used in GO, which specifies use of the words "symbiont" and "host" as the smaller and larger, respectively, of the symbiotic interactants). Among these new terms, every term that starts with "modulation" or "regulation" has two child terms, one is "positive regulation of...", and the other is "negative regulation of..." Note that these child terms are general GO terms; "position regulation," for example, includes induction, upregulation, stimulation, etc. Four diagrams (see Figures 3, 4, 5, 6) encompassing the 256 new and 38 extant GO terms explicitly depict our description of pathogenesis, with an emphasis on appressorium formation and signal transduction. More details about each step are presented in the following sections.

## Spore dispersal

Dispersal of spores is the most common process to initiate new infections [5], though direct infection by hyphae may occur. An example of the latter is the spread of ectomycorrhizal basidiomycetes in forest soils. Dispersal mechanisms can be grouped into two types: one is passive dispersal by wind, water or animal [7], and the other is active dispersal such as shooting ascospores through the boundary layer of air surrounding the fruiting body by forcible discharge [8]. Similarly, spores can be grouped into two types according to their motility. In fungi non-motile spores include sexual spores such as ascospores, rust urediniospores, sclerotia and conidiospores, while non-motile oomycete spores include oospores, sporangiospores and conidia. Motile spores with flagella, called zoospores, are ubiquitous among oomycetes and are also found in chytrid fungi [9]. Additionally, spores vary in requirements for dormancy. Some spores, such as zoospores, must encyst and differentiate to acquire qualities of dormancy before they become true spores [10]. Different spores vary in the length of the dormancy period; for example, some ascospores and oospores show extended dormancy, while others such as zoospores and ascomycete conidia are usually short-lived.

Three new GO terms including the term "GO ID 0075325 spore dispersal on or near host" were developed under the node "GO ID 0051701 ...interaction with host" to describe the mechanisms of spore dispersal. New terms describing active or passive dispersal mechanisms were placed as children of "spore dispersal on or near host" (see Figure 3).

Eight new GO terms describing spore motility were listed under the node "GO ID 0052127 movement on or near host". The term "GO ID 0075230 spore movement on or near host" is central to these eight terms with "GO ID 0075234 zoospore movement on or near host" as the principal child term (see Figure 3).

Similarly, eight new GO terms were added under "GO ID 0044408 growth or development of symbiont on or near host" to describe spore encystment. The term "GO ID 0075214 spore encystment on host" is central to these eight terms, with the term "GO ID 0075218 zoospore encystment on host" as the main child term (see Figure 3).

These 19 new terms are appropriate for annotating gene products known to be involved in spore dispersal. For example, inhibition of the vacuolar H+-ATPase by potassium nitrate causes a reduction in vacuole expulsion in zoospores of the oomycete *Phytophthora nicotianae *and leads to premature encystment [11]. Thus, H+-ATPase negatively regulates zoospore encystment and can be annotated with the new term "GO ID 0075221 negative regulation of zoospore encystment on host".

## Adhesion to the host

Adhesion of spores to the host involves physical and chemical processes [3]. Typically, when spores reach the surface of a host tissue, they attach via adhesion molecules [5]. A germination tube then emerges from the spore or the encysted zoospore (see Figure 2). From the germination tube, a growth hypha or an infection structure such as an appressorium [12-16] develops, which also becomes firmly attached to the host surface via adhesion molecules. A variety of other infection structures such as hyphopodia [17-19], haustorium mother cells [20-23], or infection cushions [24] are generated by fungal pathogens after germinating on the host surface. These all serve a common function of facilitating the pathogen's entry into the host tissue. It should be noted that the sporangia of many oomycetes may germinate directly to form an infection hypha, or else in the presence of abundant water they may differentiate, through specialized cleavage vesicles, into 10–30 zoospores that can individually disperse to initiate sites of infection [25].

Seven new GO terms under the parent, "GO ID 0044406 adhesion to host", were developed to describe in detail the biological process of adhesion to a host. The term "GO ID 0075001 adhesion of symbiont infection structure to host" is central to this section. Among the seven terms, five terms that describe adhesion of a specific infection structure, including appressorium, hyphopodium, haustorium mother cell, infection cushion, or germination tube, are children of "adhesion of symbiont infection structure to host" (see Figure 3).

To describe spore germination on or near host tissue, 16 new terms under the parent, "GO ID 0044408 growth or development of symbiont on or near host", were developed. The 16 terms cover spore germination, sporangium germination, encysted zoospore germination, and germ tube formation. The term "GO ID 0075005 spore germination on or near host" is central to this section. Major relationships among the sixteen terms are shown in Figure 3.

The 23 new GO terms in this section are useful for annotating pathogen gene products involved in adhesion to host tissue. For example, Car (cyst-germination-specific acidic repeat) proteins of the oomycete *Phytophthora infestans *are transiently expressed during germination of cysts (i.e., encysted zoospores) and during formation of appressoria, and they are localized at the surface of germlings. These proteins have considerable sequence homology to mucins and have an internal octapeptide repeat with the mucin consensus sequence TTYAPTEE. Therefore, like mucins, Car proteins should serve as a mucous cover protecting the germling and assisting in adhesion to the leaf surface [26]. Thus, the Car proteins can be annotated with the new terms "GO ID 0075226 encysted zoospore germination on or near host" and "GO ID 0075001 adhesion of symbiont infection structure to host", using the GO evidence code ISS (Inferred from Sequence or Structural Similarity).

## Signal transduction during recognition of the host

Signal transduction is an integral component of the host recognition process. Examples include protein kinase-mediated signal transduction [27], receptor-mediated signal transduction [28], G-protein coupled receptor protein signal transduction, G-protein subunit-mediated signal transduction [29], cAMP-mediated signal transduction [30], calcium or calmodulin-mediated signal transduction [31], and adenylate cyclase-mediated signal transduction [12].

In order to adequately describe signal transduction during symbiont interaction with its host, three sets of new terms were developed. Signal transduction pathways involved in the recognition between host and symbiont are generally quite extensively characterized and consequently 127 new terms were developed.

The first set of new terms is intended for annotation of host gene products that stimulate symbiont signal transduction (see Subtree 1, which includes terms under the node "GO ID 0052470 modulation by host of symbiont signal transduction pathway" in Figure 5). This set has 37 new terms. Five of these terms describing different types of signal transduction pathways are children of "GO ID 0052470" (see Subtree 1 in Figure 5).

The second set of new terms is intended for annotation of symbiont gene products that stimulate host signal transduction (see Subtree 2, which includes terms under the node "GO ID 0052027 modulation by symbiont of host signal transduction pathway" in Figure 5). This set has 36 new GO terms and has the same structure as the first set (see Subtree 2 in Figure 5). The terms in the second set are essentially the converse of the terms in the first set. For example, the term "GO ID 0075130 modulation by symbiont of host protein kinase-mediated signal transduction" in the second term set has a complementary term "GO ID 0075099 modulation by host of symbiont protein kinase-mediated signal transduction" in the first term set.

The third set of new terms is intended for annotation of symbiont gene products that stimulate symbiont signal transduction in response to the host (see Subtree 3, which includes terms under the nodes "GO ID 0051701 interaction with host" and "GO ID 0051707 response to other organism" in Figure 6). This set has 56 new GO terms. The new term "GO ID 0075136 response to host" is central to the 56 new terms. This term has 10 child terms that describe 10 symbiont responses to host organisms, such as "GO ID 0075147 regulation of signal transduction in response to host", "GO ID 0075140 response to host defense molecules", and "GO ID 0075180 regulation of transcription in response to host" etc. (see Figure 6). Eight of the 10 terms have their own child and lower level offspring terms, and each of those "response" terms has a child term such as "maintenance of symbiont tolerance to host ..." (see details in Figure 6).

The term "GO ID 0075147 regulation of signal transduction in response to host" has five children to describe different types of signal transduction, similar to the five child terms of "GO ID 0052470 modulation by host of symbiont signal transduction pathway" in the first set. Each of the five terms has child terms for positive regulation and negative regulation.

The three sets of new GO terms can be used to explicitly describe genes of signal transduction pathways involved in host recognition. For instance, the PMK1 gene of the rice blast fungus *Magnaporthe oryzae *encodes a mitogen-activated protein kinase (MAPK), which is a key component in the MAPK signaling cascade and is involved in appressorium formation and infectious growth [32]. Thus, the PMK1 protein can be annotated with the term "GO ID 0075171 regulation of MAP kinase-mediated signal transduction in response to host". Note that this gene product would not be annotated with "GO ID 0052435 modulation by host of symbiont MAP kinase-mediated signal transduction pathway" since this latter GO term is reserved to annotate host gene products. Similarly, this protein should not be annotated with "GO ID 0052080 modulation by symbiont of host MAP kinase-mediated signal transduction pathway" since PMK1 belongs to the symbiont's and not the host's signaling transduction pathway.

In addition, the modulation terms have children that describe more specific kinds of signal transduction. For example, "GO ID 0075168 regulation of protein kinase-mediated signal transduction in response to host" has a child "GO ID 0075171 regulation of MAP kinase-mediated signal transduction in response to host" (see details in Figure 6).

## Penetration into the host

Pathogens have evolved several mechanisms that include structural and/or enzymatic components in order to enter into their plant hosts [5]. Many fungi, such as *Alternaria alternata*, *Colletotrichum graminicola, M. oryzae*, *Pyrenophora teres*, and many oomycetes, such as *P. infestans *and *Phytophthora cinnamomi*, develop appressoria to directly penetrate plant cuticles [13,33-38]. An appressorium is a highly specialized structure that differentiates from the end of a symbiont germ tube. It is a swollen, dome-shaped or cylindrical organ, from which a narrow penetration peg emerges to rupture the plant cuticle and cell wall [33]. The penetration peg extends and forms a penetration hypha to penetrate through the epidermal cells and emerge into the underlying tissue [34,35]. In some instances, penetration is driven by astoundingly high turgor pressures within the appressoria [36,38]. Generation of turgor is due in part to a thick inner cell wall layer of melanin in mature appressoria. In other instances, cell wall degrading enzymes may play a primary role in or may facilitate the penetration process [39-41]. Appressoria produced by some fungi, such as rust fungi, do not penetrate directly through the cuticle, but gain entry through stomata [42].

Sixty-four new GO terms were developed to describe the biological process of penetration into the host, and they form two groups. The first group includes 43 new GO terms related to infection structures established on the outside of the host tissue, such as appressoria, hyphopodia, infection cushions, and haustorium mother cells. The second group has 21 new terms related to specialized structures that directly pierce the surface of the host, for example penetration pegs, penetration hyphae, and haustorium necks.

All of the 43 terms in the first group are children or lower level offspring of "GO ID 0052108 growth or development of symbiont during interaction with host". The core of this group is "GO ID 0075015 formation of infection structure on or near host". Twenty-eight terms in this group are related to appressorium formation. In particular, five of the 28 terms describe in detail the process of appressorium formation, namely "GO ID 0075025 initiation of appressorium on or near host", "GO ID 0075034 nuclear division during appressorium formation on or near host", "GO ID 0075033 septum formation during appressorium formation on or near host", "GO ID 0075035 maturation of appressorium on or near host", and "GO ID 0075017 regulation of appressorium formation on or near host" (see details in Figure 3).

Besides the child term "GO ID 0075016 appressorium formation on or near host", the term "GO ID 0075015 formation of infection structure on or near host" has three more detailed child terms: "GO ID 0075192 haustorium mother cell formation on or near host", "GO ID 0075187 hyphopodium formation on or near host", and "GO ID 0075183 infection cushion formation on or near host" (see details in Figure 3).

All of the 21 terms in the second group are children or lower level offspring of "GO ID 0044409 entry into host". The core of this group is "GO ID 0075052 entry into host via a specialized structure", which has three child terms related to penetration peg, penetration hypha, or haustorium neck for entry into the host (see details in Figure 4).

The 64 new terms can be used to annotate the gene products of penetration-related genes. For example, genes involved in melanin biosynthesis in the rice blast fungus, such as ALB1, RSY1 and BUF1, are required for appressorium function since mutants lacking these genes make appressoria, but are unable to penetrate susceptible rice leaves [43]; these can be annotated with the term "GO ID 0075053 formation of symbiont penetration peg for entry into host".

## Invasive growth within the host

After successful penetration, invasive hyphae are formed that ramify through the host tissue [44,45]. In some cases, special structures, such as a haustorium or an arbuscle, are formed in host cells for the symbiont to absorb nutrition [22,23].

To describe invasive growth, 15 new GO terms were developed that are children or lower level offspring of "GO ID 0044412 growth or development of symbiont within host". The term "GO ID 0075065 growth or development of symbiont in host cell" has two children, "GO ID 0052094 formation by symbiont of haustorium for nutrient acquisition from host" and "GO ID 0075066 growth or development of symbiont in host organelle". Additionally, arbuscules produced by mycorrhizal fungi are a type of structure functionally similar to haustoria, and thus "GO ID 0075328 formation by symbiont of arbuscule for nutrient acquisition from host" is a sibling of "GO ID 0052094" (see details in Figure 4).

The 15 new GO terms in this section meet the need to annotate pathogen genes that are involved in invasive growth. For example, the *MST12 *gene in the rice blast fungus *M. grisea *was found to regulate infectious growth but not appressorium formation [46]. In particular, no obvious defects in vegetative growth, conidiation, or conidia germination were observed in *MST12 *deletion mutants. Also, *MST12 *mutants produce typical dome-shaped and melanized appressoria. When inoculated through wound sites, *MST12 *mutants fail to cause spreading lesions and appear to be defective in infectious growth. As a result, *MST12 *mutants are nonpathogenic [46]. Thus, the *MST12 *gene can be annotated with the term "GO ID 0075061 formation of symbiont invasive hypha within host".

## Lesion development in the host

The eventual result of infection in most cases is lesion development. A lesion can be defined as any abnormality involving any tissue or organ due to any disease or any injury (cited from MedicineNet.com). Not surprisingly, there are many types of lesions including those caused by damage such as cold injury or insects' bites etc. It is difficult to define lesions objectively, as this requires a subjective judgment on what constitutes abnormal or damage and from what perspective, ranging for example from perturbation of a few cells to death of an entire tissue or organ. Similarly, formation of a lesion is not a specific process belonging to either the pathogen or the host and can be highly dependent on the environment. Therefore, at this time only one term, "GO ID 0009405 pathogenesis", is appropriate for genes involved in lesion formation.

## Other new GO terms

Six new terms were placed jointly under the nodes "GO ID 0006914 autophagy" and "GO ID 0044403 symbiosis, encompassing mutualism through parasitism". The term "GO ID 0075071 autophagy during symbiotic interaction" is the core of the six terms, and it has two complementary children, "GO ID 0075044 autophagy of host cells during interaction with symbiont" and "GO ID 0075072 autophagy of symbiont cells during interaction with host". The latter term has a child "GO ID 0075073 autophagy of symbiont cells on or near host surface", which itself has a lower level child "GO ID 0075074 spore autophagy during appressorium formation on or near host" (see details in Figure 3).

The six autophagy-related GO terms are applicable to describe the functions of several genes in fungal pathogens during symbiotic interaction. For example, formation of a functional appressorium in the rice blast fungus requires autophagic cell death of the conidium, which is controlled by the *MgATG8 *gene. Deletion of *MgATG8 *results in impaired autophagy, arrested conidial cell death, and a nonpathogenic fungus [14]. Thus, *MgATG8 *can be annotated with the new term "GO ID 0075074 spore autophagy during appressorium formation on or near host".

## Conclusion

Two hundred fifty-six new GO terms were developed to annotate genes or gene products involved in common pathogenic processes in fungi and oomycetes, including spore dispersal, host adhesion, recognition, penetration, and invasive growth. These new GO terms provide the opportunity to apply a standard set of terms to annotate gene products of fungi, oomycetes, and their associated hosts, as well as those of other plant-associated pathogens and their hosts. The ability to compare and contrast these annotations for widely different plant-associated microbes and their hosts, using a standardized vocabulary, will greatly facilitate the identification of unique and conserved features of pathogenesis across different kingdoms. In addition, such comparisons should provide insight into the evolution of pathogenic processes.

## Competing interests

The authors declare that they have no competing interests.
